# Monosodium glutamate as a tool to reduce sodium in foodstuffs: Technological and safety aspects

**DOI:** 10.1002/fsn3.499

**Published:** 2017-07-13

**Authors:** Hellen D. B. Maluly, Adriana P. Arisseto‐Bragotto, Felix G. R. Reyes

**Affiliations:** ^1^ Department of Food Science School of Food Engineering University of Campinas Rua Monteiro Lobato Campinas São Paulo Brazil

**Keywords:** Monosodium L‐glutamate, salty taste, sodium chloride, umami taste

## Abstract

Sodium chloride (NaCl) is the most commonly used ingredient to provide salty taste to foods. However, excess sodium in the bloodstream has been associated with the development of several chronic noncommunicable diseases. In order to limit sodium intake to levels considered safe, the World Health Organization (WHO) recommends for adults a daily intake of not more than 5 g of NaCl (less than 2 g of sodium). One of the strategic actions recommended by the Pan American Health Organization (PAHO) to reduce sodium intake is reformulation of processed foods. This recommendation indicates there is an urgent need to find salt substitutes, and umami compounds have been pointed as an alternative strategy. Like salty, umami is also a basic taste and the major compound associated to umami is monosodium L‐glutamate (MSG). The available scientific data on the toxicity of MSG has been evaluated by scientific committees and regulatory agencies. The Joint FAO/WHO Expert Committee on Food Additives and the Scientific Committee on Food of the European Commission established an acceptable daily intake (ADI) not specified, which indicated that the substance offers no health risk when used as a food additive. The United States Food and Drug Administration and the Federation of American Societies for Experimental Biology classified MSG as a Generally Recognized as Safe (GRAS) substance. In this paper, an overview about salty and umami taste physiology, the potential applications of MSG use to reduce sodium content in specific industrialized foods and safety aspects of MSG as food additive are presented.

## 1 INTRODUCTION

Over the last few years, significant changes have occurred in the global food market, which are mainly due to the growth of the population and cities, including Brazil (Trading Economics 2013). These facts may be one of the reasons for the increase in the commerce of processed foods, such as canned and ready‐to‐eat products. In 2014, marketing studies verified an increase in 9% in this sector, and the expectation in 2019 is that it could reach a retail volume of 13% of food sales (3.1 billion units of canned products) (Euromonitor International 2015).

According to a survey conducted by Brazil Food Trends 2020 (Ibope Inteligência 2010), this improvement in the market of processed foods may have partly occurred in Brazil due to changes in lifestyle and an increased demand from consumers for more convenient products, in addition to new launchings and promotional activities. The results also pointed out that sensorial quality (indicated by 23% of the consumers interviewed), along with health and wellness (indicated by 21% of the consumers interviewed), were shown by researchers as future consumption trends, which reflect the new demands of consumers. In this sense, certain types of products have been more highly valued, especially those with reduced sodium, sugar and fat content.

Sodium chloride (NaCl) is the most used ingredient to provide a general flavor in foods. In addition to its role in taste, NaCl and other food additives that contain sodium have other functions such as preservation, the acceleration of fermentation reactions and texture maintenance (Henney, Taylor, & Boon, 2010). However, excess sodium in the bloodstream has been associated with the development of various noncommunicable diseases (NCDs), also known as chronic diseases that are not passed from person to person, including hypertension and other heart problems, kidney disease, stomach cancer, and osteoporosis. To limit sodium intake at safe levels, the World Health Organization (WHO) recommends a maximum daily consumption of 5 g of salt (NaCl) for adults, which is equivalent to less than 2 g of sodium/day (WHO 2007).

Based on data published by the Brazilian Household Budget Survey (*Pesquisa de Orçamentos Familiares – POF*) carried out in 2002–2003 and 2008–2009, Brazilians have been consuming a large amount of sodium (4.7 g of sodium/day), which corresponds to more than twice the safe level (less than 2 g of sodium/day) proposed by the World Health Organization (WHO, 2007). In 2013, a study conduct by Sarno, Claro, Levy, Bandoni, and Monteiro (2013) also demonstrated that processed foods contributed to at least 25% of the sodium intake (mainly the sodium coming from NaCl addition) by middle‐class and upper‐class families of the country.

The results of these studies were used to strengthen the commitments between the Brazilian Ministry of Health (*Ministério da Saúde – MS*) and the Brazilian Association of Food Industries (*Associação Brasileira das Indústrias da Alimentação* – ABIA), which were signed in 2007 and stimulated food producers to improve the supply of health foodstuffs, including products with reduced sodium content. To develop strategies to reduce sodium intake to 2 g/day until 2020, the government renewed the agreements (BRAZIL 2007).

Among these strategies, which should also take into account the sensory quality of foods, the use of flavor enhancers such as monosodium L‐glutamate (MSG) can be considered a promising alternative with a great potential for application in the food industry (Jinap & Hajeb, 2010). MSG, which is the sodium salt of L‐glutamic acid (or L‐glutamate – dissociated form), is the most well‐known flavor enhancer used in foods, but other molecules such as nucleotides (inosinate and guanylate), other glutamate salts associated with ammonium, potassium and calcium, and other additives that contain elevated concentrations of L‐glutamate, named Natural Flavor Enhancers (NFE), such as yeast extract and products from wheat and soy fermentation, are also available in the market to enhance the flavor of foods (McGough, Sato, Rankin, & Sindelar, 2012; Yamaguchi & Takahashi, 1984).

According to the Technical Report on Sodium Content in Processed Foods published by the Brazilian Health Surveillance Agency (*Agência Nacional de Vigilância Sanitária – ANVISA*) in 2012 and 2013, some industrialized products such as soups, stocks and seasonings, instant noodles, certain snacks, processed meats and parmesan grated cheese exhibit very high sodium contents (ANVISA 2012, 2013; Sarno et al., 2009). The use of MSG and other flavor enhancers in these foodstuffs is allowed in Brazil (ANVISA, 2001) and by the Southern Common Market (MERCOSUR 2010). Thus, in this paper a review about salty and umami taste physiology, the potential applications of MSG to reduce sodium content in specific industrialized foods and safety aspects of MSG as food additive are presented, in order to contribute to the development of food products reduced in sodium without impairing the sensorial quality of the foods and the health of the population.

## 2 TASTE PHYSIOLOGY: SALTY × UMAMI TASTES

Physiological mechanisms involved in taste perception have been discussed by researchers since the beginning of the 20th century (Trivedi, 2012).

Regarding the salty taste, which is promoted by sodium chloride and potassium chloride, among other substances, the hypothesis has suggested that ion dissociation occurs when these molecules are in contact with saliva. Sodium ions, specifically cross‐specific ion channels, named ENaCs, which are in the membranes of taste cells. After the ions go in, membrane depolarization occurs, charging it positively. This depolarization increases the membrane electric potential and stimulates taste nerves, sending signals to the brain to recognize the salty taste or the presence of ions that activate the electric potential (Beauchamp & Stein, 2008; Chandrashekar et al., 2010; Geran & Spector, 2000).

In relation to the umami taste, glutamate or nucleotides get in contact with their specific G protein–coupled receptor – GPCR (mGluR4 – metabotropic glutamate receptor 4, TR1/TR3), which are present in the taste buds of the mammalian tongue (Nelson et al., 2002; Chaudhari et al. 2009). G‐proteins are high‐molecular mass compounds with different protein subtypes; each one has two subunits: Gα and Gβγ. One of the mechanisms earlier proposed suggested that substances responsible for umami activate the Gα subunit of GPCR. However, more recent investigations have shown that the main pathway for umami taste transduction seems to be related to the Gβγ subunit (Chaudhari & Roper, 2010).

After the interaction between umami‐GPCR, the βγ subunit disconnects from the Gαβγ complex and activates the enzyme phospholipase C, leading to generate IP3 (inositol triphosphate). IP3 connects with calcium channels, existing in the endoplasmic reticulum, thus stimulating the opening and release of calcium (Ca^++^) through the cytosol. Calcium ions, now in elevated concentrations in the cytoplasm, connect with TRPM5 channels in the membrane, promoting the fast influx of sodium through the cell and, consequently, depolarizing the membrane. The combined action between the elevation of calcium and membrane depolarization provokes the opening of the gap junctions, which are probably composed by pannexins (Pax1), thus promoting the release of a large amount of ATP to the extracellular space (Chaudhari & Roper, 2010; Kinnamon, 2009).

The released ATP stimulates the afferent nerve fibers, and at the same time, they excite adjacent presynaptic cells, which promote the release of 5‐HT (serotonin) and NE (norepinephrine), providing the gustative sensation in the brain. So far, it is known that if the taste buds are stimulated with umami substances, signals are reflected in the primary and orbitofrontal gustative cortex (de Araújo, Kringelbach, Rolls, & Hobden, 2003).

The interactions between the sensations of umami and salty tastes were evaluated by Yamaguchi and Kimizuka (1979). The authors verified that some intensification of the salty taste occurs when umami substances are present. The main impact is the increase in salivary secretion, smoothness and continuity of the flavor in the mouth. Nevertheless, the exact mechanisms of the gustative reception are not clear and need more investigation (Chaudhari & Roper, 2010).

## 3 APPLICATION OF FLAVOR ENHANCERS AS A TOOL TO REDUCE SODIUM CONTENT IN SPECIFIC FOOD PRODUCTS

Free glutamate occurs naturally in several foods, such as tomatoes, parmesan cheese, meats, peas, corn, mushrooms, and asparagus, among others. On the other hand, free glutamate may also be found in processed foods as a result of the use of MSG as a flavor enhancer (Yamaguchi & Ninomiya, 2000).

Since the 1950s, flavor enhancers, such as MSG, have been used in Brazil. ANVISA allows the use of MSG in several meat products, canned vegetables, food service preparations and fillings, among others. For milk products, the use is allowed only in grated cheese. For all products, the regulation specifies the use as *quantum satis* (sufficient to obtain the desired technological effect) (ANVISA 2001, 2010, 2015).

**Figure 1:**
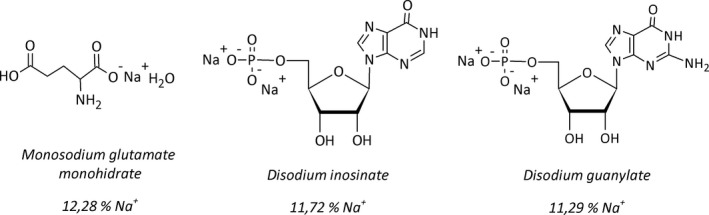
Structures and sodium content of monosodium glutamate monohydrate, disodium inosinate and disodium guanylate

In comparison to NaCl, glutamate salts such as MSG or monoammonium L‐glutamate and disodium inosinate and guanylate, have low or no sodium content (Figure 1). There is an appropriate amount of MSG for NaCl replacement with maintaining acceptance of the food. This is because an excess of MSG does not promote the umami taste and, on the contrary, could lead to an undesirable sensation (Jinap & Hajeb, 2010). The recommendation for MSG use as food additive is 0.1%–0.8% of weight, which corresponds to the amount of free L‐glutamate present naturally in tomato or parmesan cheese (Beyreuther et al., 2007). For MSG, the amount of sodium is 12.28 g/100 g, and this is 1/3 of the sodium when compared to NaCl (39.34 g/100 g). To use MSG in a homemade recipe, such as 500 g of foodstuff (rice, minced meat, etc.), a simple replacement of 1/2 teaspoon of NaCl (2.5 g) by 1/2 teaspoon of MSG (2.0 g) reduces sodium content in about 37% (Maluly, Pagani, & Capparelo, 2013).

The following sections present potential applications of MSG to reduce sodium content in specific foods.

### 3.1 Soups

Yamaguchi and Takahashi (1984) were some of the first researchers who tested different concentrations of NaCl and MSG in soups with reduced sodium content. The authors evaluated sensory panels via the consumption of *sumashi‐jiru*, a popular soup in Japan, made with dried bonito fish. The scales used by the sensory panels varied in a range of seven points that considered the amount of NaCl and palatability: from “extremely strong or palatable” (+3) to “extremely weak or unpalatable” (−3). Each panelist evaluated nine samples randomly and considered the concentration of 0.81 g/100 g of NaCl and 0.38 g/100 g of MSG as an ideal formulation. The authors verified that the reduction in the NaCl amount did not affect the palatability of the salty taste. With these concentrations, it was possible to reduce sodium content and maintain acceptability. This research suggested that to increase the palatability of reduced sodium products, MSG content should be tested at fixed concentrations while varying the levels of NaCl until finding the most appropriate combination. This is the best strategy to reduce the total sodium content in soups without influencing their palatability.

A recent study conducted by Jinap et al. (2016) investigated the acceptance of a sodium reduction in spicy soups (curry chicken and chili chicken) by Malaysian panelists, replacing NaCl with MSG. The authors verified that MSG could maintain the acceptability of the soups. The high score of acceptability was given for the soups with 0.8 g/100 g and 0.7 g/100 g of NaCl and MSG, respectively. These amounts corroborate a previous study published by Yamaguchi and Takahashi (1984), who noted that MSG could reduce the sodium content by 32.5%.

### 3.2 Stocks and seasonings

Stocks and seasonings containing NaCl are generally the main vehicles to elevate sodium consumption, according to the POFs (2002–2003 and 2008–2009) (Sarno et al., 2009, 2013).

To evaluate the acceptance of stocks and seasonings with low‐sodium content, Rodrigues, Junqueira, et al. (2014) performed a sensorial evaluation with garlic seasonings in three recipes of rice with 0%, 25% and 50% less NaCl. The seasonings were made with different proportions of NaCl, KCl (potassium chloride) and MSG. The authors verified that the panelists did not notice a strange or bad taste in the preparations, although it was reported that they contained less salt. In general, the preparations with less sodium were well accepted and the authors concluded that to these panelists, this choice could be a good alternative to reduce sodium content in homemade recipes.

Since the first industrialized stock cube was created by Julius Maggi in 1863 in Switzerland, there have been significant modifications on the formulations and consumption profile of this product. The western population is the largest consumer of stock cubes, mainly due to the convenience and flavor enhancement of the meals. However, NaCl is used in large concentrations in these products, which led to a fourth monitoring agreement to reduce sodium content, which was signed between the ABIA and the Brazilian Ministry of Health in 2013 (IDEC 2014).

MSG is commonly added to stock cubes since it can intensify the overall flavor and increase the impact, continuity and complexity of the final preparation. For stocks, the technological recommendation is 15%–25%, and for seasonings, the recommended levels are 50%‐70% (when up to 10% NaCl is used) or 8%–10% (when more than 10% of NaCl is present in the product). These percentages can vary according to the ingredients added. Good Manufacturing Practices (GMP) are suggested in order to comply with the technological recommendations and maintain the sensorial characteristics of foods (Maluly et al., 2013).

A Malaysian study conducted with commercial products identified high concentrations of free glutamate in the chicken stock powder (170.90 ± 6.40 mg/g). The authors also verified that the high concentrations were not from the addition of MSG alone but from the addition of yeast extract. These concentrations were considered safe, but slightly high for some technological protocols. Therefore, sensorial and composition analyses are suggested so that reduced sodium preparations do not exceed the technological limit of the umami taste sensitivity (Jinap & Hajeb, 2010; Khairunnisak, Azizah, Jinap, & NurulIzzah, 2009).

### 3.3 Instant noodles

Noodles are produced from wheat flour, buckwheat flour, rice flour or maize starch. These formulations can be modified according to the technology involved (Fu, 2008). Noodle dough needs NaCl, which has three different functions: gluten strengthening and elasticity, flavor and texture improvement, and the inhibition of microorganism growth and enzyme activation. Generally, 1%–3% and 8% NaCl are added to noodle formulations and *Udon* (a thick dough made with wheat flour or buckwheat), respectively (Fu, 2008). To rice flour noodles, which contain at least 7% protein, the main function of NaCl is to sustain the elasticity. Nevertheless, NaCl can reduce water penetration and increase the cooking time if the salt content is not controlled (Sangpring, Fukuoka, & Ratanasumawong, 2015).

In addition to the use of NaCl in noodle dough, salt is also added in the seasoning. The analyses of 22 samples of instant noodles available in the Brazilian market raised a great concern mainly because the results showed average levels of sodium content at 1,798 mg/100 g, with concentrations ranging between 1,435 mg/100 g and 2,160 mg/100 g when considering the overall product (dough + seasoning) and its reconstituted version (ANVISA 2012).

The umami taste has a large impact in this kind of foodstuff since the seasonings are derived from meat and vegetables stocks, which are rich in glutamate, inosine and guanosine monophosphate. In the food industry, the use of umami substances in seasonings is extensive, and their recommended concentrations are 10–17 g/100 g for MSG or monoammonium glutamate (MAG), 0.5–0.7 g/100 g for disodium inosinate, and 0.3–0.7 g/100 g for the mixture of I + G (disodium inosinate + guanylate) (Maluly et al., 2013).

In addition, it is suggested here that the technological recommendations concerning the use of flavor enhancers not be exceeded. Thereby, to reduce sodium in these products, the first approach is to create strategic government campaigns and warn the population about the reward of flavor variety in order to avoid food monotony, which will depend on consumption education and food behavior modifications (Henney et al., 2010).

### 3.4 Meat products

In general, meat products contain high sodium contents. NaCl is added for preservation purposes since it can prevent the growth of some pathogenic bacteria, extend the shelf life, ensure safety and texture, and make the products softer (mainly lean meat) and with better flavor. Table 1 shows some processed meat products consumed in Brazil and their sodium contents, according to the Brazilian Food Composition Table (TACO, 2011).

**Table 1:** Processed meat products and sodium content

Processed meat products	Sodium content (mg/100 g)^a^	Portion (g)^b^	Sodium content/portion (mg)
Formed ham	81	30	283
Jerked beef, cooked	1443	30	433
Jerked beef, raw	5875	30	1763
Beef burger, raw	869	80	695
Beef burger, fried	1252	80	1002
Beef burger, grilled	1090	80	872
Chicken sausage, raw	1126	50	563
Chicken sausage, fried	1374	50	687
Chicken sausage, grilled	1351	50	676
Pork sausage, raw	1176	50	588
Pork sausage, fried	1432	50	716
Pork sausage, grilled	1456	50	728
Bologna sausage	1212	40	485
Cooked ham with fat covering	1021	40	408
Cooked ham without fat covering	1039	40	416
Salami	1574	40	630
Belly pork, raw	50	100	50
Belly pork, fried	125	100	125
Salty cod fish, braise	1256	60	754
Sardine, canned	666	60	400

a Values from TACO (2011).

b Calculation determined through the RDC nº 359, 12/23/2003 (ANVISA, 2003).

In an attempt to minimize the problems associated with high sodium consumption, diverse protocols have been developed without compromising sensorial quality and the safety of meat products. Thus, Ruusunen, Simolin, and Puolanne (2001) verified an intensification of flavor when MSG, disodium inosinate (IMP) and guanylate (GMP) nucleotides were added to Bologna sausage formulations. The authors also demonstrated that even MSG added alone enhanced the sensorial quality of the products. The salty taste sensation was improved in sausages with MSG.

Quadros, Rocha, Ferreira, and Bolini (2015) evaluated the sensorial profile of a fish (Mackerel) hamburger with reduced sodium. The hamburgers were formulated with whole minced fish after removing residues via washing, such as hemoglobin and soluble proteins, which may interfere in the flavor and shelf‐life of the preparations. The concentrations of 1.5 g/100 g and 0.75 g/100 g of NaCl associated with 0.3 g/100 g of MSG were used in the different formulations. The results showed that MSG did not interfere directly with the final scores, but it contributed to the increase in acceptance scores among products containing 0.75 g NaCl/100 g (50% reduction).

### 3.5 Snacks

In addition to potato chips, different kinds of snacks have emerged in the market, such as tortilla chips in Mexico and the US, *pretzels* in Italy and Germany, and popcorn, hazelnuts, nuts and seeds, and meat products, such as jerky in the US. Among Brazilian commercial snacks, other than potatoes, tortillas and extruded snacks, there are some nuts, such as peanuts and Brazil nuts.

TACO (2011) provided the sodium content of some snacks. In salted roasted almonds only traces were found; concentrations at 167 mg/100 g were verified in roasted and salty cashew nuts, whereas 16 mg/100 g were reported in Brazil nuts. ANVISA (2012) reported levels of 1,092 mg/100 g in salty cassava flour biscuits, 779 mg/100 g in corn‐extruded snacks, 741 mg/100 g in salty crackers and 624 mg/100 g in potato chips.

Brazilian customers are beginning to look for healthy snacks, such as those made with vegetables and with reduced sodium and fat content (Barbosa, Madi, Toledo, & Rego, 2010). Consequently, the food industry has been trying to differentiate their products in this competitive market. Thus, flavor enhancers, as MSG, have been applied in snacks to reduce sodium content. However, the total substitution of NaCl by flavor enhancers is not possible. Nevertheless, these substances are used to harmonize the salty taste during taste perception. MSG stimulates the salivation and intensifies other flavors, such as aromas from certain herbs used in these products (Ainsworth & Plunkett, 2007).

To reduce NaCl in snacks, the technological recommendation of MSG is up to 0.5 g/100 g. This is mainly due to the interactions with the salty taste and its property of covering‐up the residual bitter taste promoted by NaCl substitutes, such as potassium chloride (Yamaguchi & Kimizuka, 1979).

Attempts to use MSG in potato chips were made to reduce oil uptake and salt content. A solution containing NaCl (0.5 g/100 g) and MSG (0.03–0.3 g/100 g) was used in six different tests in vacuum conditions. Consequently, a transfer mass phenomenon was produced by the impregnation of the solutes into the potato and the loss of water from the pores. The entrance of the two solutes into the pores, partially substituting the water lost in the process, reduced the evaporated water bubbles inside the tissue. The fried potatoes, with 19% less water, consumed less heat flow (160–165°C) to change the vapor state, whereas a larger amount of sensitive heat was required to act in the dry matter, which favors the decrease in the oil flow into the pores. This could also lead to the decrease in acrylamide formation, which is desirable from a health and safety point of view and to the formation of a crunchy layer, which is desirable from a sensorial point of view. Furthermore, NaCl and MSG were used in low concentrations when compared to the usual conditions, reducing the sodium content (Silvera, 2013).

### 3.6 Milk products

The direct addition of MSG is generally not applied in cheeses (ANVISA, 2015). Nevertheless, some researchers performed specific sensorial analyses to test flavor enhancer applications in mozzarella and cream cheese (Rodrigues, Gonçalves, Pereira, de Deus Souza Carneiro, & Pinheiro, 2014; Silva, de Souza, Pinheiro, Nunes, & Freire, 2013).

Three formulations of brines used for the preparation of Mozzarella cheese were evaluated: A (without reduced NaCl – 300 g/L); B (25% reduced NaCl – 225 g/L + 64.6 g/L of KCl + 40.2 g/L of MSG); C (50% reduced NaCl – 150 g/L + 43 g/L of KCl + 160.8 g/L of MSG). The formulations B and C resulted in a 30 and 54% sodium reduction, respectively. These reductions were obtained due to the diffusion coefficients of the salts in brines. After Time‐Intensity (TI) evaluation of the salty taste and Temporal Dominance of Sensations (TDS) tests, it was possible to verify that the modifications of the sodium content did not affect the palatability significantly; however, a lower salty taste sensation was reported. The reduced sodium formulations were widely accepted, and the authors discussed that the use of MSG and other flavor enhancers in formulations containing KCl are crucial for avoiding bitter or metallic residues (Rodrigues, Gonçalves, et al., 2014).

Cream cheese was investigated by Silva et al. (2013). Different types of NaCl substitutes were tested including KCl, magnesium and calcium chloride, calcium and potassium lactate, and potassium phosphate. The authors found that KCl provided the highest salting equivalence when compared to NaCl, while MSG had a lower equivalence of salt. The partial substitution of NaCl by MSG intensified the salty taste according to the TDS test, giving the continuity of the sensation for 8 min. Beyreuther et al. (2007) reported that flavor enhancers impart the umami taste and boost other flavors. Thus, researchers need to pay attention to their self‐limiting addition of those food additives.

## 4 SAFETY ASPECTS OF MONOSODIUM GLUTAMATE

The available scientific data on the potential toxicity of MSG included studies on acute, subchronic and chronic, toxicity, as well as studies on teratogenicity and reproductive toxicity, in different animal species such as rats, mice, dogs, and rabbits. A detailed discussion on the results of those studies was reported by Reyes, Areas, and Maluly (2013).

### 4.1 Toxicological aspects

#### 4.1.1 Metabolism and pharmacokinetics

After ingestion, glutamate is absorbed by the cells of the gastrointestinal tract and is catabolized in the cytosol and mitochondria by the transamination reaction under the action of various enzymes present in the stomach, intestines, and colon. One of the products of this catabolism, α‐ketoglutarate, can enter the tricarboxylic acid cycle, releasing energy (ATP) and carbon dioxide. Other metabolic products include lactate, glutathione, glutamine, alanine, and various other amino acids (Burrin & Stoll, 2009).

Most of the glutamate present in foods (up to 95%) is metabolized by the first‐pass effect and is used as an energy source by the enterocytes of the intestinal mucosa, whether it was added as a food additive or was naturally present in food (Reeds, Burrin, Stoll, & Jahoor, 2000). Therefore, even after the ingestion of large amounts of protein in the diet, the glutamate levels in plasma are low due to its rapid metabolism in the intestinal mucosa cells. The ingested glutamate that is not metabolized in the gastrointestinal tract enters the hepatic portal circulation and is metabolized in the liver, generating energy via the Krebs cycle or being converted into urea for excretion in urine (Burrin & Stoll, 2009).

The food components may also reduce the plasma concentration of glutamate when compared to the oral administration of the substance in water, especially if the food is rich in metabolizable carbohydrates. These carbohydrates provide pyruvate as a substrate for glutamate in the mucosal cells, so more alanine is formed and less glutamate reaches the portal circulation (Stegink, Filer, & Baker, 1987).

#### 4.1.2 Toxicity

In tests performed with rats and mice, very low acute toxicity was verified after the oral administration of glutamate, with an LD_50_ (lethal dose that kills 50% of the animals studied) ranging from 10 to 22.8 g/kg bw (JECFA 1988; Walker & Lupien, 2000).

JECFA (1988) evaluated subchronic and chronic toxicity studies conducted in rats and mice and that included the reproductive phase. Data showed that long‐term exposure to MSG, when administered in the diet at up to 4.0%, did not elicit any specific adverse effects in the evaluated animals. Similar results were observed in 2‐year studies conducted with dogs who received 10% MSG in the diet.

Reproductive adverse effects and teratogenicity were not observed even when females were fed high doses of glutamate, indicating that the maternal diet does not increase the exposure of the fetus and neonate by transplacental transfer or by the nursing milk, respectively (JECFA, 1988).

### 4.2 Safety of monosodium glutamate consumption

#### 4.2.1 Estimated intake of glutamate from foods

The estimate of human exposure to glutamate from foods, for risk assessment purposes, will depend on the characteristics of the diet and should consider both the natural sources of the amino acid (glutamate bound to protein and free glutamate) and the use of its salts as flavor enhancers in processed foods.

Although the information regarding the amount of glutamate ingested by humans are scarce in the literature, the average intake of the forms naturally present in foods (as part of the protein or in the free form) in a Western diet has been estimated to be approximately 10 g/day, which is equivalent to 0.17 g/kg bw, assuming a 60 kg person. For glutamate used as a food additive in Western diet, the estimated intake ranged from 0.3 to 0.5 g/person/day (0.005–0.008 g/kg bw) for average consumers and reached 1 g/person/day (0.017 g/kg bw) for high consumers. In Asian countries, the glutamate intake from the addition of MSG or other salts is higher, ranging from 1.2 to 1.7 g/person/day (0.02–0.03 g/kg bw) for average consumers and can reach 4 g/person/day (0.07 g/kg bw) for high consumers (Beyreuther et al., 2007).

Thus, the available data suggest that glutamate intake from the use of MSG as food additive is much lower when compared to the estimated values from glutamate naturally present in foods.

#### 4.2.2 Monosodium glutamate consumption and the Chinese Restaurant Syndrome

The safety of MSG has been widely discussed since the Chinese Restaurant Syndrome (or the complex of symptoms related to MSG) was first described by Kwok (1968), which reported a set of signs and symptoms including pain in the neck or head and weakness and palpitations after consuming Chinese food, or more precisely, food containing MSG. Also, some authors have linked the intake of MSG to other symptoms such as asthma, atopic dermatitis, urticaria, respiratory difficulty, and tachycardia (Allen, Delohery, & Baker, 1987; Gann, 1977; Ratner, Eshel, & Shoshani, 1984; Van Bever, Docx, & Stevens, 1989). Nevertheless, studies, including double blind, placebo‐controlled investigations, performed to confirm the relationship between the intake of MSG and the development of the previously described symptoms show no plausible association, or the existence of a sensitive subpopulation to MSG, since self‐identified sensitive individuals to MSG failed to show reproducibility of different symptoms (Geha et al., 2000; Yang, Drouin, Herbert, Mao, & Karsh, 1997).

These observations, together with the limitations that make it impossible to assume the existence of any positive association demonstrated in some studies, indicate that it is unlikely that the consumption of MSG is involved in the onset of the symptoms associated with the Chinese Restaurant Syndrome. In addition, scientific evidence does not suggest the participation of MSG in the onset of asthma, urticaria, angioedema or rhinitis (Williams & Woessner, 2009).

### 4.3 Opinions of the scientific committees and regulatory agencies regarding the use of monosodium glutamate as a food additive

The safety of MSG has been evaluated by scientific committees and regulatory agencies, including the Joint FAO/WHO Expert Committee on Food Additives (JECFA), the Scientific Committee on Food (SCF) of the European Commission, the United States Food and Drug Administration (FDA), and the Federation of American Societies for Experimental Biology (FASEB). A summary of the evaluations is presented below.

#### 4.3.1 Joint FAO/WHO Expert Committee on Food Additives (JECFA)

The safety of MSG as a food additive was evaluated by JECFA in its 14th (1971), 17th (1974) and 31st meetings (1988). In the first evaluation, an ADI of 0–120 mg/kg bw, expressed as L‐glutamic acid, was established by the Committee and excluded children under 12 weeks of age (FAO/WHO, 1971). This ADI was maintained in the second evaluation in 1973, but the restriction of MSG in infant food was withdrawn in view of the fact that adverse effects were not observed in neonate animals at the recommended levels of glutamic acid salts for use as a food additive (FAO/WHO, 1974). In its latest assessment performed in 1987, based on the available scientific data (exposure, chemical, biochemical, toxicological, etc.) at the time of the evaluation, an ADI “not specified” was established by the Committee for MSG and the ammonium, calcium and potassium salts, which indicated that the total glutamate daily intake (derived from its use as a food additive and its natural presence in foods) does not represent a risk to human health (FAO/WHO, 1988).

#### 4.3.2 Scientific Committee for Food (SCF)

An evaluation performed by the Scientific Committee for Food of the European Commission (SCF, 1991) resulted in findings similar to those reported by JECFA, that is, an ADI “not specified” can be attributed to MSG.

In 1995, the European Parliament established a limit of 10 g/kg (1.0%) for the use of MSG as food additive, added alone or in combination with other glutamic acid salts such as potassium, calcium, ammonium, and magnesium. The decision remained valid at the meeting in 2008, and the limit was maintained (European Parliament and the Council of the European Union, 1995, 2008).

#### 4.3.3 Food and Drug Administration (FDA) and the Federation of American Societies for Experimental Biology (FASEB)

In 1958, the United States FDA classified MSG as a substance “Generally Recognized as Safe” (GRAS). The agency maintained this classification when it reassessed the available data on the substance 20 years later. However, in the 1980s, due to concerns raised by some published studies showing associations between the consumption of MSG and induction of adverse health effects, the FDA asked FASEB a complete review of the scientific data regarding the safety of this food additive. FASEB, which was formed by a group of independent scientists, completed its evaluation in July 1995 (FASEB, 1995). According to the FDA, the results were consistent with the assessments previously conducted by the other committees (JECFA and SCF), confirming both the safety of MSG when used as a food additive at the recommended levels (0.1%–0.8% in food) and the lack of evidence that chronic exposure to MSG causes health problems in the general population.

#### 4.3.4 Brazilian Health Surveillance Agency *(ANVISA)*

In Brazil, ANVISA takes into consideration the safety assessments conducted by JECFA and US FDA. Thus, no restrictions were established for MSG as a food additive, which is used in accordance to Good Manufacturing Practices (GMP) in an amount sufficient to achieve the desired technological effect (“*quantum satis*”) (ANVISA, 2010).

## 5 FINAL COMMENTS

Considering the available data in the scientific literature, in addition to the information provided by the flavor enhancer industry, we could verify the use of umami substances as a strategy to reduce sodium in different foodstuffs (processed and homemade foods) without affecting the perception of saltiness and, therefore, contributing to the wellness and safety of the population. Many applications evaluated showed promising results, especially in those products with elevated sodium contents such as processed meat.

Despite concerns about the toxicity potential of MSG raised by some studies, regulatory agencies have demonstrated the safety of use of this food additive through toxicity assessments and randomized double blind, placebo‐controlled studies. An ADI “not specified” or GRAS status has been allocated to glutamate and its salts, meaning that it can be used as a food additive in the necessary amount to achieve the desired technological effect. Nonetheless, in the European Union, a use limit of 10 g glutamate/kg of food has been established.

Other strategies, such as the use of nucleotides (IMP and GMP) and NFEs, could also be useful for enhancing products with reduced sodium content. The combination of different substances in a formulation could generate a larger impact in flavor continuity due to their synergistic effect when added at recommended concentrations to maintain the desirable flavor without exceeding the sensorial and technological limits.

Sensorial and physicochemical tests are always recommended to obtain higher quality products while respecting the preferences of the consumers and the lifestyles of the modern life.

## CONFLICT OF INTEREST

The first author, Hellen D. B. Maluly, is a voluntary researcher and has been working as a Food Industry Consultant. She has interest in food additives that improve flavor in foods and ingredients that provide health benefits, both in the field of safety and technological application.

The other authors do not have conflicts of interest.
